# Comparison of small symptomatic and asymptomatic abdominal aortic aneurysms based on computational fluid dynamics analysis

**DOI:** 10.1097/MD.0000000000027306

**Published:** 2021-10-01

**Authors:** Zhijun Zhou, Biyun Teng, Yu Zhao, Zhe Wang

**Affiliations:** Department of Vascular Surgery, First Affiliated Hospital of Chongqing Medical University, Chongqing, China.

**Keywords:** abdominal aortic aneurysm, computational fluid dynamics analysis, intra-luminal thrombus, symptomatic small abdominal aortic aneurysms, wall shear stress

## Abstract

**Background:**

The purpose of this study was to compare the hemodynamic parameters of symptomatic and asymptomatic abdominal aortic aneurysms (AAAs) to explore the risk factors for AAA rupture.

**Methods:**

We conducted a retrospective analysis of 26 patients with symptomatic small AAAs and 60 patients with asymptomatic small AAAs. Computational fluid dynamics methods were used to compare hemodynamic characteristics between the symptomatic and asymptomatic groups and to evaluate risk factors for the occurrence of symptomatic AAAs.

**Results:**

The maximum diameters in the symptomatic and asymptomatic groups were 49.7 ± 4.94 mm and 48.4 ± 4.55 mm, respectively. Wall shear stress values at turbulent flow regions in the symptomatic and asymptomatic groups were 0.0098 ± 0.0084 Pa versus 0.0174 ± 0.0068 Pa, respectively. Shear stress values at the site with maximal blood flow impact force in the symptomatic and asymptomatic groups were 1.13 ± 0.466 Pa and 2.04 ± 0.42 Pa, respectively. The areas of the intra-luminal thrombus in the section with the maximum diameter in the symptomatic and asymptomatic groups were 952.19 ± 413.53 mm^2^ versus 646.63 ± 296.88 mm^2^, respectively.

**Conclusion:**

The wall shear stress in the symptomatic group was lower than that in the asymptomatic group.

## 1 Introduction

Abdominal aortic aneurysm (AAA) is a type of macrovascular disease with an extremely high risk of serious complications and mortality. Once an aneurysm ruptures, the mortality rate can reach 70% to 90%.^[1]^ In prior studies, 3% to 15% of treated aneurysms have been described as symptomatic.^[2]^ The currently recommended surgical indications include a maximum diameter of the AAA greater than 55 mm in men or 50 mm in women and rapid growth (>10 mm/year).^[3–5]^ However, the mortality and serious complication rates among patients with symptomatic AAA are significantly higher than those among patients with asymptomatic AAA. Moreover, perioperative mortality is twice as high in patients with symptomatic AAA than in those with asymptomatic aneurysms.^[5–7]^ To date, no precise method is available to assess hemodynamic factors for symptomatic small AAAs.

Blood flow plays a vital role in the pathogenesis of AAA and is converted into biosignals that cause morphological changes in the AAA, which counteract and cause changes in blood flow. Blood flow drives the remodeling or growth of aneurysms according to the pathological mechanism and is an important factor affecting the outcome of AAA.^[8]^ In recent years, computational fluid dynamics (CFD) has been used to evaluate the risk of AAA rupture, thus providing important pathophysiological information for blood flow in AAAs and facilitating prediction of the potential risk of AAA rupture and associated risk factors.^[9–11]^ However, many AAA ruptures occur in areas with a large intraluminal thrombus (ILT)^[12]^ and reduced wall shear stress.^[13]^ However, few studies have examined the correlation between CFD and small AAA. The CFD method can be used to analyze the local hemodynamic factors of small AAAs, helping better predict the outcome and formulate corresponding treatment plans.

The purpose of this study was to analyze blood flow in an actual subrenal AAA with a small diameter based on CFD and to compare the blood flow structure and local hemodynamic factors such as wall shear stress and ILT area between symptomatic and asymptomatic AAAs with a small diameter to predict risk factors associated with the occurrence of symptomatic small AAAs.

## 2 Materials and methods

### 2.1 Ethical review

This study was approved by the Ethical Committee of the First Affiliated Hospital of Chongqing Medical University (Approval No. 2017-179). The patients were informed and signed the informed consent.

### 2.2 Clinical data

From January 2016 to March 2018, patients with AAA who were consecutively admitted to our center were selected. A statistical analysis of the patient information, medical history, and blood pressure was performed. Patients with the following conditions were excluded: suprarenal AAA, unavailability of computed tomography angiography (CTA) imaging data, the maximum diameter of aneurysms in male patients >55 mm, the maximum diameter of aneurysms in female patients >50 mm, duration of symptoms more than 60 days, ruptured AAA, abdominal aortic dissection, and lower extremity arterial embolism.^[14]^ Ultimately, a total of 26 patients with symptomatic small AAA and 60 patients with asymptomatic small AAA were included, and we retrospectively analyzed their data (Table 1), symptomatic AAA means back pain, abdominal pain, hip joint pain, tenderness in the aneurysm, or bloating aneurysm dilatation, or new-onset vascular thrombosis.^[15]^

**Table 1:** Patient demographics.

	Asymptomatic N = 60	Symptomatic N = 26	*P* value
Age (yrs)	72.5 ± 7.0	71.8 ± 8.5	.701
Gender (M/F)	46/14	12/14	.006
CAD^∗^	17	7	.893
Diabetes	11	7	.368
Hypertension	19	12	.199
Smoking	21	9	.973
Drinking	18	8	.943
BMI^†^	23.7 ± 1.5	23.2 ± 2.2	.208

∗ CAD = coronary artery disease.

† BMI = body mass index; weight (kg)/length^2^ (cm).

### 2.3 CTA data collection and 3D CFD modeling

The CTA imaging data (slice thickness: 1 mm) of all patients at the time of admission and follow-up were downloaded from the imaging workstation of our hospital and stored in digital imaging and communications in medicine format. Using the professional workstation, the CTA imaging data were imported in digital imaging and communications in medicine format into the Mimics 19.0 medical image processing program (Materialise Inc, Belgium) to reconstruct a 3D model of the target vessel, and the 3D model was processed for mesh optimization and fairing processing using Mimics’ image optimization software 3-matic 11.0. The number of elements of a fine mesh ranged from 4,577,883 to 821,275, whereas the number of elements in coarse mesh ranged from 938,712 to 1,230,951. When the maximum relative errors of the wall shear stress magnitudes were <5% between a fine and coarse mesh, the coarse mesh was considered satisfactory and adopted for simulation.

### 2.4 Definition of boundary conditions

The entrance boundary was set to a level of 20 mm above the neck of the AAA. The inlet blood flow rate, inlet pressure, and outlet pressure were set to 0.18 m/s, 140 mm Hg, and 0 mm Hg, respectively.^[8]^ The blood can be considered as an incompressible Newtonian fluid with a density of 1.05 g/cm^3^ and a viscosity of 0.0035 Pa/s. The vascular wall is considered a rigid wall without displacement. The Reynolds number of each patient was calculated according to the Reynolds formula: Re = 2ρuR/μ, where ρ is the blood density, u is the inlet velocity, R is the vascular radius, and μ is the kinematic viscosity. The corresponding mean Reynolds number based on the inlet diameter varied from 539 to 867. The governing equation used in numerical simulations was the 3-dimensional unsteady Navier-Stokes equation.

### 2.5 Calculation of the values

The Fluent module in the advanced finite element analysis software package ANSYS 15.0 (ANSYS, USA) was used for calculations. Under the above-mentioned boundary conditions, the hemodynamic parameters of 26 patients in the symptomatic group and 60 patients in the asymptomatic group were calculated by CFD simulation.

### 2.6 Calculation of the ILT area

**Figure 1:**
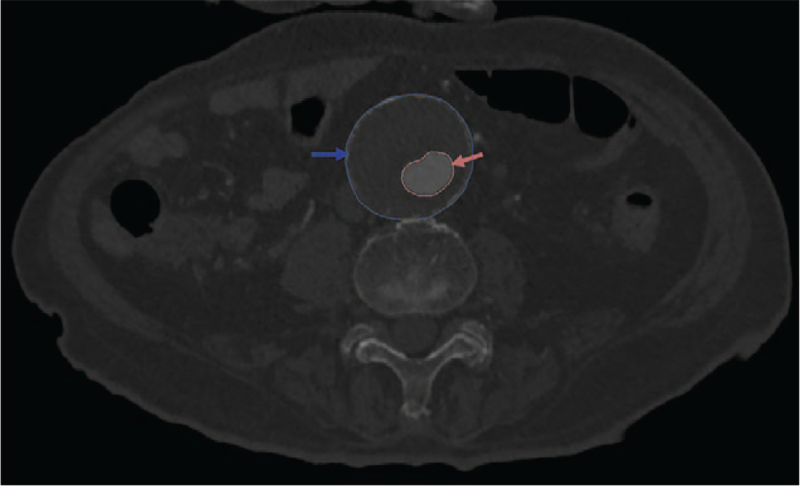
The section of the abdominal aortic aneurysm with the largest diameter. The region within the red line represents blood flow (red arrow). The region between the blue line and the red line represents the intra-luminal thrombus (blue arrow).

The area of the AAA, blood flow area, and ILT area at the site of the aneurysm rupture were measured using the Mimics 19.0 area measurement function (Fig. 1).

### 2.7 Statistical analysis

SPSS 23 (IBM SPSS Inc., Chicago, IL) was used for data analysis. Quantitative data were compared using a paired sample *t* test. Qualitative data were compared using a chi-square test. A value of *P* < .05 was considered statistically significant. Quantitative data are expressed as the mean ± SD (X ± S).

## 3 Results

### 3.1 Demographics

No statistically significant differences were found between the 2 groups concerning age, hypertension, diabetes, chronic obstructive pulmonary disease, stroke, use of cardiovascular and anti-inflammatory medication, peripheral vascular disease, or current smoking. The results showed no significant difference in maximum systolic blood pressure between the asymptomatic and symptomatic groups (symptomatic: 140.3 ± 15.8 mm Hg, asymptomatic: 136.8 ± 20.42 mm Hg).

### 3.2 Maximal diameter of the AAA

The maximal diameters were 49.7 ± 4.94 mm and 48.4 ± 4.55 mm in the symptomatic and asymptomatic groups, respectively.

### 3.3 Comparison of ILT areas

**Figure 2:**
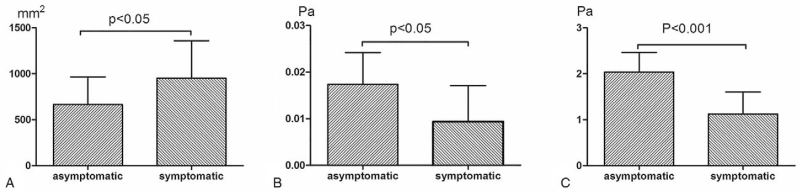
(A) The area of the section with the maximal diameter of the thrombus in the 2 groups; (B) The shear stress in the section with the maximal diameter of the thrombus in the 2 groups; (C) The wall shear stress at the site with the maximal blood flow impact force in the 2 groups.

The area at the level with the largest diameter of the ILT in the symptomatic group was compared with that in the asymptomatic group. The area in the maximal diameter of the ILT in the symptomatic group was 952.19 ± 413.53 mm^2^, and that in the 60 patients in the asymptomatic group was 646.63 ± 296.88 mm^2^ (Fig. 2A). Thus, the thrombus area was significantly greater in the symptomatic group than in the asymptomatic group (*P* < .05).

### 3.4 CFD analysis showing changes in shear stress within aneurysms

CFD analysis was performed on the symptomatic and asymptomatic groups, and the results showed that the wall shear stress was unevenly distributed within the AAA. The shear stress at the level of the maximal diameter in the symptomatic group was 0.0098 ± 0.0084 Pa, and the wall shear stress at the site with the maximum impact area of the blood flow was 1.13 ± 0.466 Pa. In the asymptomatic group, the shear stress at the site with maximal blood flow impact force was 2.04 ± 0.42 Pa, and the shear stress at the level of the maximal diameter was 0.0188 ± 0.0068 Pa. The shear stress at the site with the maximal blood impact force was significantly different from that in the symptomatic group (Fig. 2B, C) (*P* < .001). Hemodynamics plays an important role in the development of AAA.

### 3.5 Blood flow characteristics within aneurysms

**Figure 3:**
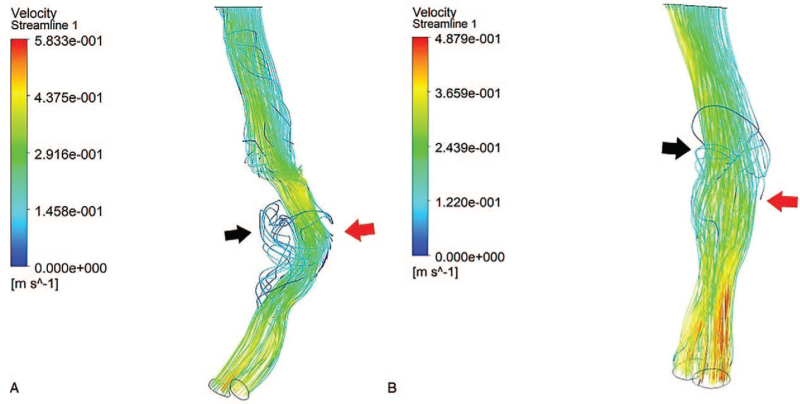
(A) A symptomatic small abdominal aortic aneurysm; (B) An asymptomatic small abdominal aortic aneurysm. The black arrow indicates a low-velocity turbulent region, and the red arrow indicates the site with maximal blood flow impact on the abdominal aortic wall. The blue region indicates a low-speed value, and the red region indicates a high-speed value.

The blood flow structure in the lumen of AAA is complicated and includes a dominant blood flow channel and a low-speed vorticity flow zone in the aneurysm lumen (Fig. 3). Due to the expansion of the aneurysm lumen, part of the blood flow in the aneurysm lumen is relatively slow. According to the flow velocity diagram, the blood flow in the dominant blood flow channel is fast, while the blood flow in the turbulent flow region is slower. The blood flow direction in the turbulent region is inconsistent with that of the dominant blood flow channel.

## 4 Discussion

Assessment of the hemodynamic factors for symptomatic small AAA is essential to reduce the mortality in patients with AAA.^[7]^ Accurately predicting the occurrence of symptomatic small AAA can help to understand the progression of AAA and to develop a precise medical treatment plan. However, effective prediction is a challenging task. The occurrence, development, and rupture of AAA are the consequence of the interaction of biomechanical dynamic changes between blood flow and the blood vessel wall, which leads to the degradation and remodeling of the extracellular matrix.^[13]^ Unlike finite element analysis, which is more applicable to static structural aortic wall mechanics, CFD is a numerical method that provides a dynamic prediction of aortic flow characteristics, such as wall shear stress. CFD analysis can be used to identify accurate risk factors. Although the method is complicated, CFD can be used to analyze the local hemodynamic factors of AAA to more accurately predict their risk,^[16]^ compared to methods using the maximum diameter, morphological parameters, and serum markers. Of course, other factors affect the rupture of AAA. The higher the arterial blood pressure, the easier it is for AAA to rupture,^[17]^ this may be related to the rapid increase in blood pressure, resulting in excessive pressure on the AAA, leading to the rupture of the AAA. In addition, related studies have shown that calcification is also related to the rupture of AAA.^[18]^ At the same time, women and smoking are also the rupture factors of AAA.^[19]^

This study described the blood flow structure and wall shear stress in 26 patients with symptomatic small AAA and 60 patients with asymptomatic small AAA. Meanwhile, the area of the thrombus in the lumen was calculated to predict the prognosis of patients with AAA. This is the first study to examine the risk factors of small AAA using CFD combined with intra-luminal thrombosis data.

**Figure 4:**
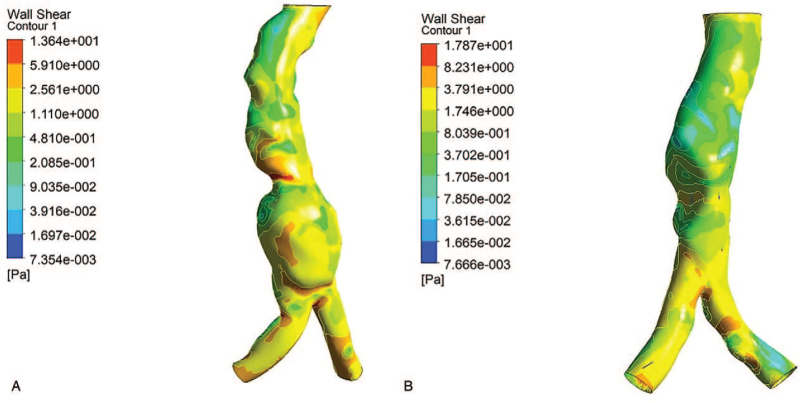
(A) Wall shear stress in a symptomatic small abdominal aortic aneurysm; (B) Wall shear stress in an asymptomatic small abdominal aortic aneurysm.

Our data showed no significant difference in blood pressure or chronic diseases between the 2 groups. In the lumen of AAA, the blood flow velocity was low, the blood flow structure was characterized by turbulent flow, and the wall shear stress was lower than that in the surrounding regions (close to zero) (Fig. 4). Intra-luminal thrombosis was observed. According to a comparison of the 2 groups of patients, the blood flow structure in the symptomatic group was significantly more disordered than that of the asymptomatic group. The wall shear stress values at the site with the maximal diameter and the site with maximum blood flow impact force were significantly lower in the symptomatic group than in the asymptomatic group. The degree of ILT was significantly greater in the symptomatic group than in the asymptomatic group.

High wall shear stress can promote the initial formation of an aneurysm.^[20,21]^ After the formation of an AAA, turbulent blood flow, decreased blood flow velocity, and decreased wall shear stress within the AAA can promote activation of platelets in the proximal turbulent region. The activated platelets can adhere to the walls with low shear stress to promote intra-luminal thrombosis. These changes can result in the formation of a barrier between the endothelial layer of the abdominal aortic wall and blood flow-induced shear stress. This barrier causes a significant reduction in the shear stress directly on the wall of the AAA. Moreover, the ILT promotes the expansion and degeneration of the aortic wall, which suggests that an interaction between low wall shear stress and ILT accumulation can promote AAA growth.^[22–24]^ Aortic walls not covered with ILT presented a dense collagenous matrix with differentiated smooth muscle cells, whereas those covered with ILT contained de-differentiated apoptotic cells.^[25]^ Hypoxia occurs in AAA wall regions with a thick ILT. This may lead to increased local inflammation, the formation of new blood vessels in these areas, and weakening of the local vascular wall.^[26]^ These affect the stability of the AAA wall and lead to the expansion and rupture of the AAA.^[27]^

### 4.1 Limitations

The main limitation of our study was the small sample size.

### 4.2 Future directions

In terms of directions for future research, further work could expand the sample size.

## 5 Conclusions

Blood flow velocity, low wall shear stress, and high ILT are risk factors to assess the risk factors associated with small AAA and help vascular surgeons develop appropriate regimens.

## Acknowledgment

We acknowledge and appreciate our colleagues for their valuable efforts and comments on this paper.

## Author contributions

**Conceptualization:** Zhijun Zhou, Yu Zhao, Zhe Wang.

**Data curation:** Zhijun Zhou, Biyun Teng.

**Formal analysis:** Zhijun Zhou, Zhe Wang.

**Funding acquisition:** Yu Zhao, Zhe Wang.

**Investigation:** Zhijun Zhou, Zhe Wang.

**Methodology:** Zhe Wang.

**Project administration:** Yu Zhao.

**Resources:** Zhijun Zhou, Yu Zhao, Zhe Wang.

**Software:** Zhijun Zhou.

**Supervision:** Yu Zhao, Zhe Wang.

**Visualization:** Zhijun Zhou, Biyun Teng, Zhe Wang.

**Writing – original draft:** Zhijun Zhou, Biyun Teng, Zhe Wang.

**Writing – review & editing:** Zhijun Zhou, Biyun Teng.
